# FISH Oracle 2: a web server for integrative visualization of genomic data in cancer research

**DOI:** 10.1186/2043-9113-4-5

**Published:** 2014-03-31

**Authors:** Malte Mader, Ronald Simon, Stefan Kurtz

**Affiliations:** 1 Center for Bioinformatics, University of Hamburg, Bundesstrasse 43, 20146 Hamburg, Germany; 2 Department of Pathology, University Medical Center Hamburg-Eppendorf, Martinistrasse 52, 20246 Hamburg, Germany

**Keywords:** Genomic data, Integrative visualization, Cancer genomics, Web server, GenomeTools, ICGC, TCGA, TTC28

## Abstract

**Background:**

A comprehensive view on all relevant genomic data is instrumental for understanding the complex patterns of molecular alterations typically found in cancer cells. One of the most effective ways to rapidly obtain an overview of genomic alterations in large amounts of genomic data is the integrative visualization of genomic events.

**Results:**

We developed FISH Oracle 2, a web server for the interactive visualization of different kinds of downstream processed genomics data typically available in cancer research. A powerful search interface and a fast visualization engine provide a highly interactive visualization for such data. High quality image export enables the life scientist to easily communicate their results. A comprehensive data administration allows to keep track of the available data sets. We applied FISH Oracle 2 to published data and found evidence that, in colorectal cancer cells, the gene TTC28 may be inactivated in two different ways, a fact that has not been published before.

**Conclusions:**

The interactive nature of FISH Oracle 2 and the possibility to store, select and visualize large amounts of downstream processed data support life scientists in generating hypotheses. The export of high quality images supports explanatory data visualization, simplifying the communication of new biological findings. A FISH Oracle 2 demo server and the software is available at http://www.zbh.uni-hamburg.de/fishoracle.

## Background

The availability of high-throughput technology in genomics, such as microarrays and more recently *Next Generation Sequencing* (NGS), led to many interesting projects in all areas of the life sciences, the most well-known of which probably are the *Encyclopedia of DNA Elements* (ENCODE) project [1], the *1000Genomes* project [2], *The Cancer Genome Atlas* (TCGA) [3], and the *International Cancer Genome Consortium* (ICGC) [4]. These are complemented by thousands of smaller projects [5]. These projects delivered large sets of data which has spawn the development of many new and efficient methods and software tools to analyze the data. However, due to the exponential decrease in the costs to produce the data and the fact that computer hardware costs decreased less dramatically, the bottleneck in genomics research shifted from data generation to data analysis.

Most of the methods and tools for analyzing genomics data focus on the initial steps of the analysis, such as identifying a set of highly expressed genes in a microarray or RNA-seq study or predicting mutation events in a population, given a set of reads mapped to a reference genome. Such analysis steps reduce the data to be analyzed further by at least two orders of magnitude. However, the complexity and diversity of the results, here called *downstream processed data*, requires well-thought methods to integrate the data in a common model, such that all queries relevant for the specific research questions can efficiently be answered. Sometimes it suffices to produce a ranked list of genomic events, e.g. the most differentially expressed genes or highly consistent mutations supported by data from a given set of samples. In many cases, for a given set of samples, one wants to obtain an overview of all genomic events in a specific genomic location. In this situation, the integrative visualization of specific downstream processed data is usually a necessary and often sufficient mode of analysis.

The many software solutions for the integrative visualization of genomic data can be divided into three main categories: 

1. *generic genome browsers* including the *Ensembl genome browser*[6], the *UCSC genome browser*[7], *GBrowse*[8] and *JBrowse*[9].

2. genome browser for short read data (*NGS browser*) including *Trackster*[10] (part of the *Galaxy Visualization Framework*[11]), *Artemis*[12], *GenomeView*[13], *Savant2*[14], and the *Integrative Genomics Viewer* (IGV) [15]. For an in depth review of generic genome browsers and NGS browsers see [16,17].

3. *special purpose* software for the visualization of downstream processed genomic data, such as the *Gaggle genome browser*[18] and *myKaryoView*[19].

*myKaryoView* is a web application to visualize personal genomics data provided via the *Distributed Annotation System* (DAS) protocol [20] by services like 23AndMe [21]. The data can be viewed at different resolutions ranging from whole chromosomes to regions of a few hundred base pairs. The Gaggle genome browser unifies the visualization of several kinds of data ranging from microarray probes and short read data to generic features. It employs the *Gaggle framework*[22] which provides interfaces to a rich set of bioinformatics applications.

All these tools are useful for the task they were developed for. However, in our opinion, they are all lacking some important functionality for the convenient integrative visualization of downstream processed data, namely a flexible filter system for the data to be displayed, high quality image export for explanatory data visualization and communication as well as a powerful data administration to cope with the ever increasing amounts of data to be integrated.

In our previous work [23] we have described the first version of the FISH Oracle software (*FISH Oracle 1*, in the sequel) for the visualization of copy number data, build on top of a fast visualization engine and *Asynchronous JavaScript and XML* (AJAX) technology offering an interactive visualization and convenient export of visualizations in high quality formats. Here, we describe *FISH Oracle 2*, a *special purpose* software tool for the integrative analysis of cancer genomics data. It stores different kinds of downstream processed data from multiple samples in a single database. A powerful search interface allows to interactively filter the data to be displayed with respect to different criteria. *FISH Oracle 2* is able to simultaneously display different data sets, thus simplifying their comparison. Filter and display options can be changed on the fly. A comprehensive data administration allows to keep track of the data stored in the database. *FISH Oracle 2* provides significant advances and many new features, compared to *FISH Oracle 1* (see Additional file 1: Table S1 for details).

In two case studies we show how to apply *FISH Oracle 2* to different kinds of genomic data from published ICGC and TCGA projects and exemplify its capability for flexible and effective visual data exploration. For example, in the data from the TCGA project (see second case study), the visualization revealed evidence that the gene *TTC28* may be inactivated in two different ways in colon and rectal tumor tissue, a finding that has not been published before.

## Methods

For storing data, creating the user interface, handling the client/server communication, and visualizing the data, *FISH Oracle 2* employs the same well-established software tools and libraries already used in its predecessor *FISH Oracle 1*. These tools and libraries and their use have extensively been described in a previous publication [23]. This section briefly outlines the latest modifications of and additions to the methods used in *FISH Oracle 1*. Additional file 1: Figure S5 shows a summary chart of the most important components of *FISH Oracle 2*.

The data visualized in *FISH Oracle 2* comes from two sources, namely the *FISH Oracle database* and the Ensembl database. To simplify the description, we use the term Ensembl as a synonym for the Ensembl database if not stated otherwise. Annotations are fetched from Ensembl, either by accessing a remote server (e.g. at the European Bioinformatics Institute) or a local server storing a verbatim copy of the original database. For efficiency reasons, we recommend the latter mode of access.

In *FISH Oracle 1* we used the Java Application Programming Interface (Java API) to Ensembl. As this interface is no longer supported, we had to modify the parts of the software which access Ensembl. One choice would have been to employ the fully supported Perl API to Ensembl. However, this would have added another programming language in addition to C and Java and would also require additional effort in interfacing Perl with AnnotationSketch [24] (our visualization library, written in C). To avoid these complications, we developed our own C API to Ensembl. As we only use a subset of the data stored in Ensembl, this turned out to be a reasonable decision. Our C-API supports multiple versions of Ensembl, providing access to chromosome bands and high level gene data.

The data specific to *FISH Oracle 2* is stored in the *FISH Oracle database*, which has been extended to accommodate different kinds of processed genomic data originating from NGS and microarray experiments as well as user related data. The latter kind of data is generated whenever the user decides to store configurations of the search menu or other display configurations to refine them later or to reproduce a certain view on the data.

The data in the *FISH Oracle database* can be accessed either via a C API or via a Java API. To retrieve and visualize data we use the C API, which is built on top of the *GenomeTools* software [25], in particular the persistent graph based storage functionality for genome annotations, supporting MySQL and SQLite databases. A complete C-based solution has proven to be much faster and more robust than a combined C- and Java-based solution as used in *FISH Oracle 1*. The Java API to the *FISH Oracle database* is solely used for data administration (e.g. data import) and tabular display of data.

## Results

*FISH Oracle 2* consists of three main parts: *data visualization*, *data administration*, and *application administration*. These are described in the following three subsections. We further present two detailed case studies showing how to use *FISH Oracle 2* in the analysis of two publicly available cancer genome data sets.

### Data visualization

**Figure 1:**
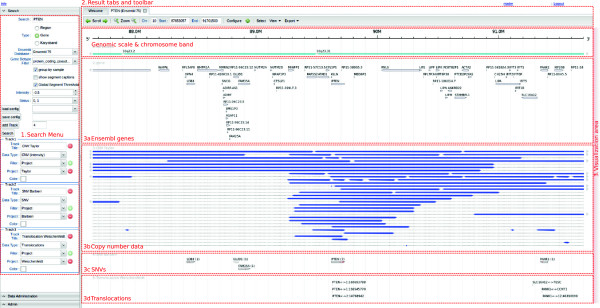
**Main window of FISH Oracle 2.** Main window of *FISH Oracle 2* focusing on the 10q23 locus which includes the tumor suppressor gene *PTEN*. Part (1) shows the search menu, which, in the displayed case, is used for searching a gene by its name. Part (2) consists of the toolbar showing different navigation and configuration options. Part (3) consists of the visualization area with the chromosome band below the genomic scale and four further tracks, one for Ensembl genes (in this case genes corresponding to Ensembl version 75 are displayed, indicated by the caption of the tab), one for CNVs (deletions in our case), one for SNVs and one for translocations, all displayed in default colors. All CNVs with a segment intensity value of less than -0.5 are shown. The SNV track displays several genes containing between one and seven SNVs followed by seven translocations in the next track. Each track shows a different data set (here obtained by microarrays and NGS), but all data refer to prostate cancer tissue samples. The most frequently occurring copy number changes form a minimal common region including the gene *PTEN*. Furthermore, *PTEN* is associated with seven SNVs in one dataset and three translocations in eleven samples of EO-PCA. The characteristic minimal common region derived from three different kinds of genomic events highlight the gene *PTEN*.

*FISH Oracle 2* allows users to query the *FISH Oracle database* in a variety of ways. It enables an interactive visual data exploration of large amounts of different kinds of genomic data in the context of genome annotations offering a chromosomal resolution ranging from a few base pairs to whole chromosome view. Several easily configurable filters can be applied to select data with specific attributes, which are specified in the search menu on the left side of the main window of *FISH Oracle 2* (Figure 1). The selected data is displayed in the main window using one of the following four visualization elements, each of which has an optional caption providing additional information about the displayed genomic entity.

A *segment* is a horizontal rectangle of arbitrary length and fixed height with an associated numerical attribute. A segment is most appropriate for representing data on copy number variations (CNVs), i.e. genomic amplifications or deletions annotated with intensity or status values, which could be computed by programs such as DNACopy [26] and PENNCNV [27], respectively. Segments can also be used to represent expression values. A segment caption consists of the sample name the data was derived from.

A *SNV-element* is a vertical line of fixed height at a specific position. It is most appropriate for displaying *single nucleotide variations* (SNVs). The caption of a SNV-element shows the identifier of the sample the SNV was derived from. If a SNV is located within a known gene, then the corresponding gene name is displayed in the caption. The number following the gene name indicates how many SNVs have been found within the boundaries of this gene.

A *translocation-element* is a vertical line of fixed height at a specific position representing the first of two chromosomal positions. A visualization of the genomic area covering the second position can be obtained by a mouse click on the translocation-element. A translocation-element is most appropriate for displaying interchromosomal *translocations* with two chromosomal break points. The caption of a translocation-element contains the two chromosomal positions of the translocation break points separated by a double arrow. If the break points overlap with a gene, then the name of the gene is displayed, instead of the genomic position.

**Figure 2:**
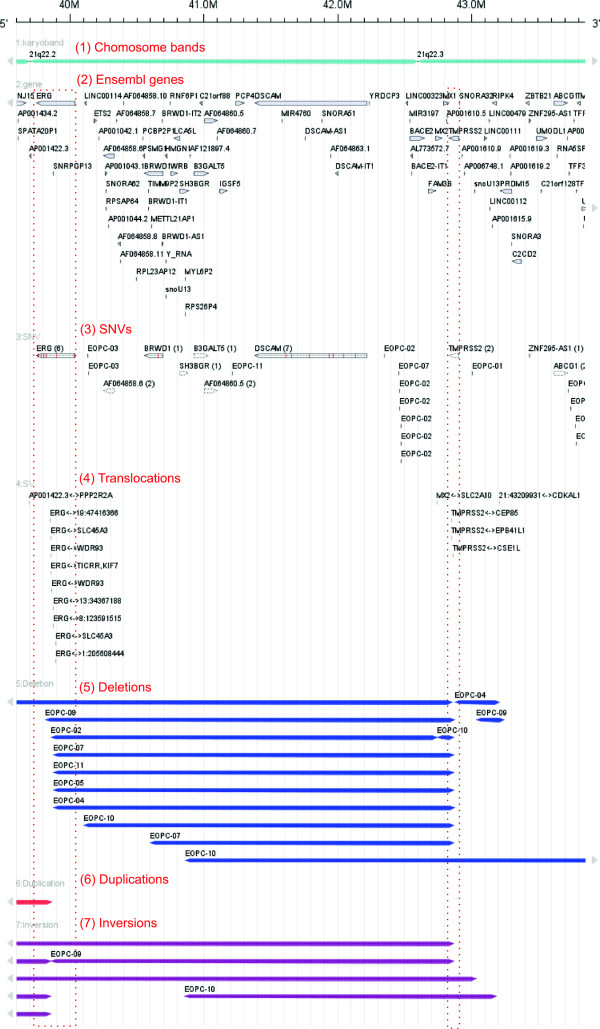
**Genomic aberrations in EO-PCA at the 21q22 (ERG) locus.** This region focuses on the fusion gene *TMPRSS2:ERG*[28]. In addition to the chromosome band (1) and gene annotations (2), five data tracks (dataset "Weischenfeldt" in the demo application) are displayed. Track (3) shows all SNVs including six in *ERG* and two in *TMPRSS2*. Track (4) displays inter chromosomal translocations, nine of which include *ERG* and three of which affect *TMPRSS2*. Track (5) shows several cases of interstitial deletions leading to the *TMPRSS2:ERG* fusion. The chromosomal ranges of *TMPRSS2* and *ERG* overlapping with deletions are indicated by thin dotted red boxes. Track (6) shows one duplication and track (7) shows seven inversions. Tracks (5), (6) and (7) visualize generic entities, i.e. data specified by genomic coordinates only.

Genomic entities (including processed data or self-defined entities) not fitting into any of the previous three categories can be visualized by *generic elements*, i.e. rectangles of fixed height and arbitrary length. A generic element only requires the specification of genomic coordinates, consisting of a chromosome identifier as well as a start and end position of the genomic entity, and an optional name. Examples for the use of generic elements can be found in Figure 2 and Additional file 1: Figure S1.

At the top of the search menu, the user has several options to request the visualization of data for a specific genomic region. This can either be done by specifying genomic coordinates (chromosome identifier, start and end position of the region) or a gene name or the identifier of a chromosome band. In the latter two cases, the displayed chromosomal region is determined as described in [23]. The chromosomal region refers to the Ensembl database chosen in a selection menu. *FISH Oracle 2* supports all Ensembl databases from version 54 (last version referring to the NCBI36 human genome assembly) up to the current version, which is version 75, as of March 2014. A mouse click on the visual elements opens a popup window or a new tab, displaying additional information like the exact chromosomal position, associated attributes and links to external databases including *RefSeq Genes*[29], the *Single Nucleotide Polymorphism Database* (dbSNP) [30] and the *Database of Genomic Variants* (DGV) [31].

Different options controlling how segments are displayed can be chosen via check boxes. First of all, segments can be displayed either unordered or grouped by sample. In the latter case, every line of a track (i.e. a logical collection of lines) displays segments of the same sample. This ensures that the data between different samples within a track is comparable. If the user chooses to not order the segments, they are positioned in a compact layout, which is often advantageous for a large number of segments to be displayed. In particular, peaks of minimally common genomic regions of recurring segments are easier to spot, compared to the mode which groups the segments by sample. Figure 1 (part 3b) show an example for grouped and Additional file 1: Figure S2 (track (2) and (3)) show an example for unordered segments.

If many segments are displayed, their captions can clutter the visualization. On the other hand, captions often contain useful information which supports the interpretation of results. Therefore, the user can choose whether segment captions should be displayed or not.

To select segments according to their intensity value, one chooses an intensity threshold value. Negative threshold values define an upper bound and positive threshold values define a lower bound. Only segments with intensity values smaller than the upper bound or larger then the lower bound are displayed. To select segments according to their status, one chooses from a selection of possible status values using a drop down menu. Only segments with status from the chosen set of status values are displayed.

Sometimes one wants to compare data sets which have been generated by different experimental methods. To make such data comparable, *FISH Oracle 2* allows to specify different intensity threshold values or status values for each individual dataset, thus allowing, for example, to display amplifications and deletions on top of each other in different tracks. In other cases it may be necessary and more convenient to choose the same threshold or selection of status values for all datasets to be displayed. To simplify this, the user can choose a threshold for intensities or a selection of status values to be valid for all tracks (global segment threshold).

The track system of *FISH Oracle 2* enables the user to create an arbitrary number of tracks, each displaying different kinds of data, as chosen by the appropriate *data type* choice box for the track. The following data types can be chosen from: CNV, SNV, translocations and generic data, each corresponding to a visualization element as described above. Depending on the chosen data type, the user can set further options to filter the data with respect to certain attributes. For each data type there are at least two options: (1) The data can be filtered by project names, i.e. a set of projects can be chosen and all data available for any of these projects will be selected. (2) The data can be filtered by tissue, i.e. only data for the specified tissue will be selected. In addition to the generic filters described here, there are specific filters to select mutation data with respect to quality, somatic status, confidence and the SNP tool that was used to predict the mutations. To easily identify a track, the user specifies a *track title* to be displayed in the visualization.

To further customize the visualization, *FISH Oracle 2* allows to choose different colors for the tracks. Custom colors override the default colors for the different kinds of data. There are two default colors for segments with intensity values: blue represents segments with negative values and red depicts segments with positive values.

A mouse click on the start button visualizes the selected data in a new tab with a toolbar at the top. Below the toolbar a genomic scale appears followed by data displayed in different tracks. The first two tracks are fixed and show the identifiers of the chromosome bands of the chosen chromosome, followed by the gene annotations according to the selected Ensembl version. The number of tracks that follows is unlimited and depends on the choices of the user, as described above. In particular, each track specified by attribute selections in the search menu triggers the visualization of corresponding actual data in a track in the main window.

The toolbar on top of the visualization enables the user to navigate over the chromosome or to zoom into or out of the chromosome. The exact positions of the displayed chromosomal region are shown in the toolbar. The configuration button opens a configuration menu for the currently displayed visualization. This menu allows to change the search and visualization settings while the user is browsing the genome. The select button allows the user to mark interesting regions within the main window by a rectangle with red boundaries. A mouse click on the *View* button opens a new browser tab showing the currently displayed genomic region within the Ensembl or UCSC browser. This is useful in cases where, besides the gene annotation displayed in *FISH Oracle 2*, additional genomic features available in Ensembl or in the UCSC-database are of interest. The *FISH Oracle 2* visualization can be exported in different custom size high quality image formats, namely PDF, scalable vector graphics (SVG), PostScript and portable network graphics (PNG).

Due to the many possible choices in the selection menu, a configuration of *FISH Oracle 2* resulting in a specific visualization can get fairly complex. To simplify the process of completely reproducing a configuration, it is possible to save it in the *FISH Oracle database* and to later load it into the search or configuration menu when needed. The saving of configuration options should not be confused with a bookmark, available in other genome browsers. While a bookmark allows a user to recover a specific genomic location on the chromosome with minimal effort, saved search configurations simplify restoring a specific selection of position independent filter and visualization options.

### Data administration

The *data administration* section of *FISH Oracle 2* provides a detailed view of the different kinds of genomic data stored in the *FISH Oracle database*. Additionally, it allows to import and delete data, to assign data to different data sets and to manage access permissions to data for groups of users.

The data in *FISH Oracle 2* is structured as follows: All data is associated with a *study*, identified by a unique study name. A study may contain data of the four different data types specified above. It refers to the kind of tissue (coming with optional attributes), the data refers to. A set of studies defines a *project*. Studies may be included in different projects. Access to data is defined on the project level, that is, each project can be accessed by certain user groups. A registered user can access data belonging to a project if he/she is included in the corresponding user group.

The data administration comprises three parts: data import, project administration and study administration.

For the *data import*, the files containing the data of interest are uploaded to the server. For each file, the user specifies the kind of data as well as the study (existing or new), the data belongs to. For each new study, the user specifies a project, a tissue, a platform that was used to produce the data and a genome assembly to which the data refers.

The import section of the *FISH Oracle 2* offers the user the choice to either import all data from all uploaded files at once (batch mode) using given meta information for all studies or to perform the input interactively, file by file. In the latter case, the software requests the required information for each file from the user. Options already set can be reviewed and changed. With any study, additional general attributes, e.g. biological or pathological meta information about the tissue can be associated.

Importing large sets of files via the web interface can become tedious. Therefore *FISH Oracle 2* comes with a command line tool called *Fish-Oracle-importer*, to perform an automatic batch import. It parses a tab delimited file specifying, in each line, the paths to the data files to be uploaded as well as the corresponding meta information, as described above. Each data file along with the meta information defines a complete study, with the filename serving as study name. In this way, a large number of studies including their meta information can be automatically created in *FISH Oracle 2*.

The *project administration* provides an overview of all projects in the *FISH Oracle database*, including the studies belonging to a project and the user groups having access to it. New projects can be created, old ones can be deleted and studies can be assigned to projects. The permission to access a project can be granted or revoked from user groups.

The *study administration* gives a more detailed view over the studies imported into *FISH Oracle 2* as well as the data contained in the studies.

### Application administration

This part of *FISH Oracle 2* allows to administrate meta information like registered users, user groups and the identifiers for platforms, tissues and databases. Registered users can be activated or their password can be reset. Groups can be created and users can be assigned to groups.

### Case study 1 ICGC: Early onset prostate cancer

*The International Cancer Genome Consortium* (ICGC) [4] coordinates a global network of large scale cancer studies to obtain a comprehensive description of genomic, transcriptomic, and epigenomic changes in the 50 most important human tumor types. These include prostate cancer, the most frequent malignant tumor in males. In one project, prostate cancers (PCA) developing below the age of 50 years (early onset PCA, EO-PCA for short), are analyzed in order to dissect early molecular alterations connected to PCA development. The results of this study suggest that, on the molecular level, EO-PCA represents a distinct subset of PCA, characterized by particularly frequent structural rearrangements (SRs) including the TMPRSS2:ERG gene fusion [32]. *FISH Oracle 2* was among the tools used for detecting potentially important tumor relevant regions in EO-PCA.

Here we present *FISH Oracle 2* visualizations for publicly available data from the EO-PCA study. The data was retrieved from the supplemental material of [32].

Figure 2 shows the human chromosome 21q22 locus including the *TMPRSS2* and *ERG* genes and the alterations detected in eleven EO-PCAs. The *FISH Oracle 2* visualization demonstrates that structural rearrangements, such as deletions with breakpoints inside the *TMPRSS2* and *ERG* genes, are the dominant events in this region. These interstitial deletions, as shown in track 5 (deletion) result in the *TMPRSS2:ERG* gene fusion, which brings the *ERG* transcription factor under the control of the constitutively activated *TMPRSS2* promoter. While *ERG* is usually not expressed in prostate cells, the gene fusion results in massive overexpression of *ERG*. Several studies have shown that *ERG* expression in prostate cells is associated with activation of potential oncogenic pathways, including *WNT* and *TGF- β* signaling [33-35].

Interestingly, some deletion breakpoints are located outside of *TMPRSS2* and *ERG*, and other types of structural rearrangements, including duplications (track 6), inversions (track 7), and translocations (track 4) are also frequently present. The impact of such SRs for prostate cancer biology is currently not understood, but a reasonable hypothesis is that they develop – like the *TMPRSS2:ERG* gene fusion – as a consequence of highly active androgen receptor (AR) signaling. It has been shown that strong AR signaling (a hallmark of EO-PCA) induces chromatin movements resulting in chromatin crossover and eventually structural rearrangements in conjunction with erroneous double strand breakage repair [36]. In contrast, single nucleotide variants (SNVs, track 3 of Figure 2), indicating possible oncogenic mutations, are rare in this region. This observation is consistent with earlier reports suggesting that prostate cancer is characterized by frequent SRs rather than SNVs [37]. As shown here, *FISH Oracle 2* allows for simultaneous visualization of all types of such SRs in a single image. Such a comprehensive view on all relevant data is instrumental for understanding the complex patterns of molecular alterations typically found in cancer cells.

Additional file 1: Figure S1 depicts alterations found in the human chromosome 10q23 locus, specifically at the *PTEN* tumor suppressor locus. The minimal region of overlapping deletions (track 5), as visualized by *FISH Oracle 2*, includes *PTEN* as well as a few adjacent genes. *PTEN* is highlighted as a gene with many deletions in the different tumor samples. This is consistent with previous findings [38,39]. The translocations (track 4) indicate a complex rearrangement involving chromosomes 1 and 10, resulting in a disruptive breakage and loss of the function of *PTEN* (compare figure two b in [32]). This finding was of particular importance as it suggests that intragenic breakage may be an alternative mechanism of *PTEN* inactivation in prostate cancer.

**Figure 3:**
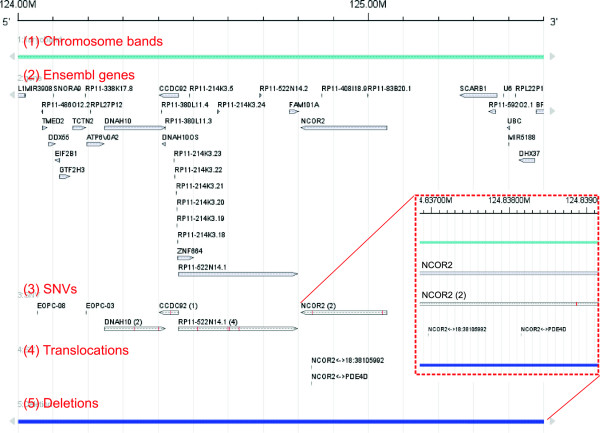
**Genomic aberrations in EO-PCA at the 12q24 locus.** Genomic aberrations in EO-PCA at the 12q24 locus. This region focuses on the androgen-receptor interacting gene *NCOR2*. In addition to the chromosome band (1) and gene annotations (2), three data tracks (datasets "TCGA_COAD" and "TCGA _READ" in the demo application) are shown. Track (3) shows all SNVs, two of which target *NCOR2*. Track (4) shows two translocations, one of which leads to a gene fusion of *PDE4D* and *NCOR2*. Track (5) shows one deletion (generic features) covering *NCOR2*. The insert in the red box magnifies a part of the *NCOR2* gene, including SNVs and two translocations.

Figure 3 exemplifies how *FISH Oracle 2* facilitates the identification of genes that are recurrently affected by genomic alterations of different types. The nuclear receptor corepressor 2 (*NCOR2*) gene has five alterations in the 11 EO-PCA cases. These include a large deletion accompanied by a disruptive translocation in case EO-PCA06, resulting in complete inactivation of *NCOR2*. Given the functional involvement of *NCOR2* in chromatin remodeling and AR-dependent transcription control [40,41], this finding already qualifies *NCOR2* as an interesting candidate for further investigations. The presence of mutations in two additional tumors (track 3) as well as another case of translocation (track 4), further supports the hypothesis that the *NCOR2* gene is relevant for prostate cancer biology. Note that some alterations (i.e. translocations and the 3 ^′^-mutation of *NCOR2*) seem to be located very closely together when viewed at a high zoom level. However, the flexible zooming capabilities of *FISH Oracle 2* quickly reveals that these events are actually not co-localized.

### Case study 2 TCGA: Colon and rectal cancer

*The Cancer Genome Atlas* (TCGA) is a U.S. network for large-scale cancer studies to reveal relevant molecular genomic changes in human cells, leading to the development of different tumors [3]. As of March 2014, data from 29 different tumor types has been generated and is available online at the *TCGA Data Portal*[42].

Here we show *FISH Oracle 2* visualizations of some interesting genomic regions that were found in the latest study of colon and rectal cancer [43]. The data was separated into two distinct data sets, one containing samples from colon and one from rectal tissue. The SNP data was retrieved from the supplemental material of [43] and from the *TCGA Data Portal*[42].

Additional file 1: Figure S2 shows a chromosomal overview of CNV data. Two tracks are used to separately show the CNV data from colon and rectal samples. This allows to quickly compare the data from the two cancer types, revealing similarities and differences. For example, focal deletions of *RBFOX1* (16p13 locus) are present in both cancer types, while *WWOX* (16q24 locus) deletions are only visible for colon cancers.

**Figure 4:**
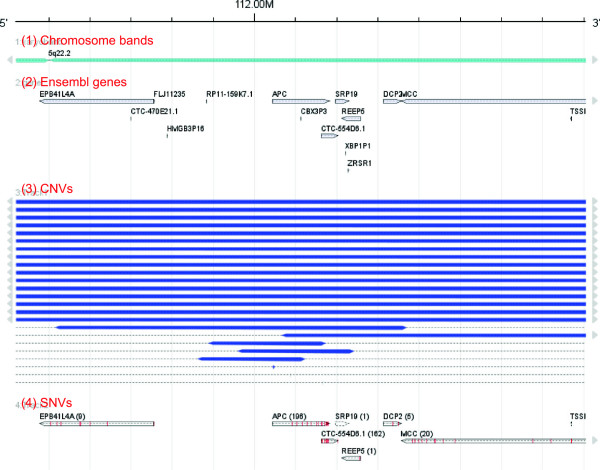
**Abundant deletions and mutations in the genomic locus of the gene APC.** In addition to the chromosome band (1) and gene annotations (2), two data tracks (datasets "TCGA_COAD" and "TCGA_READ" in the demo application) are shown. Track (3) shows CNV intensity data displaying deletions at a threshold of -0.5. CNV segments are grouped by sample. Track (4) shows many SNVs, 196 of which affect the gene *APC*.

Another interesting finding in this dataset was a minimally overlapping region of a deletion at the human chromosome 5q22 region, highlighting the genomic position of the *APC* tumor suppressor gene (Figure 4). Note the high prevalence of mutations of *APC* (in total there are 196 SNVs in the two datasets) [43]. The *APC* locus is another example that a simultaneous visualization of deletion and mutation tracks in *FISH Oracle 2* supports detection of "typical" tumor suppressor genes following the "second hit" sequence of inactivation [44].

In colorectal cancer, inactivation of the *fragile histidin triad* (*FHIT*) gene in the human chromosome 3p14 region does not seem to involve SNVs, but frequent deletions, the majority of which are small. A possible but not yet validated hypothesis is that this visual pattern represents the particularly high degree of genomic instability at the *FHIT* locus, which is co-localized with the fragile site *FRA3B*, one of the most instable regions of the human genome [45] (Additional file 1: Figure S3).

Additional file 1: Figure S4 gives an example of the capability of *FISH Oracle 2* to identify target genes of amplifications. In particular, the displayed human chromosome 11p15 locus with frequent amplifications in samples from colorectal cancer is shown. *INS* and *TH* are the only genes inside the minimal commonly amplified region, and *INS* has been described as the functionally relevant amplification target gene [43].

**Figure 5:**
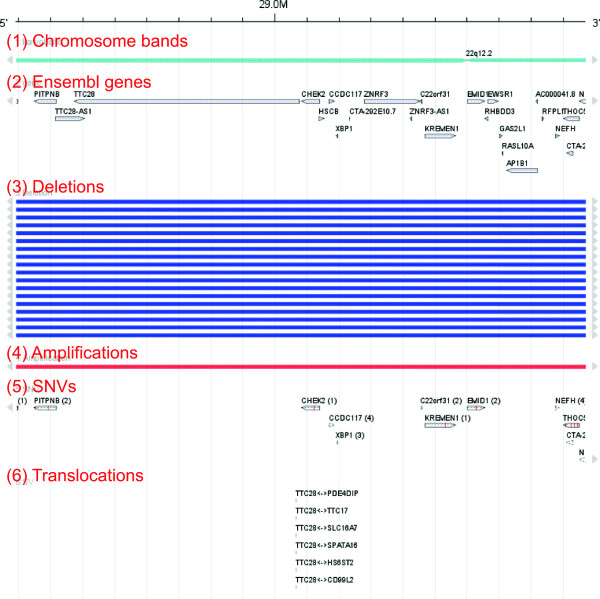
**Genomic aberrations in colon and rectal tumors at the 22q12 locus.** This region focuses on the frequently translocated gene *TTC28*. In addition to the chromosome band (1) and gene annotations (2), four data tracks (datasets "TCGA_COAD" and "TCGA_READ" in the demo application) are shown. Track (3) and (4) show CNV intensities in form of deletions (3) and amplifications (4) at a threshold of -0.5 and 0.5 respectively. Track (5) shows SNVs and track (6) shows translocations. The gene *TTC28* is affected by six translocations all of which result in a gene fusion.

Figure 5 indicates the presence of biallelic hits in the human chromosome 22q12 locus, which includes the gene *TTC28*. A comparison of the case IDs reveals that in five of six cases with a translocation, also a deletion is present, resulting in complete inactivation of *TTC28*. Interestingly, no mutations of *TTC28* were found in this study, further supporting the hypothesis that a combination of deletion and breakage may be an important – and probably alternative – mechanism for complete gene inactivation in neoplasia. This is the first report suggesting a biallelic inactivation of *TTC28* in colon and rectal cancer, thus complementing recent findings of [43], describing *TTC28* as frequently hit by translocations. The combination of tracks displaying SNVs and translocations massively facilitates the identification of areas in the genome harboring potentially interesting candidate genes. With the increasing application of the mate pair sequencing technique, it is likely that a multitude of novel translocations will be detected, some of which might represent a common second hit in areas with large deletions.

## Discussion

We have developed *FISH Oracle 2*, a flexible web server for visualizing different kinds of processed genomic data in context of genome annotations. *FISH Oracle 2* supports interactive as well as batch import of data. A convenient user interface allows for fine grained selection of the data to be displayed, and a powerful visualization engine quickly produces state-of-the-art images. These display different data types such as CNVs, SNVs, generic data and translocations. While the first three data types are shown in a similar way as in other software tools, *FISH Oracle 2* provides a unique visual representation of translocations, which includes the positions, possibly the affected gene, and an interactive component to optionally display the genomic area containing the opposite breakpoint.

All data in *FISH Oracle 2*, including user defined parameter settings for the selection of specific subsets of the data, are stored in a relational database. This guarantees data consistency, fast retrieval of the data and reproducibility of results. The organization of the data into projects and studies (which can belong to one or more projects) and the unified view on the data via a data management component allows to easily keep track of large amounts of data of different kinds. The high quality of the visualization and the flexibility of the software allows life scientists to quickly derive interesting hypotheses about candidate cancer genes which are affected by genomic events such as copy number variations, single nucleotide variations or structural variations.

In two case studies we have exemplified this process by applying *FISH Oracle 2* to data from the ICGC and the TCGA project. For example, the visualization of data in the second case study provides additional clues that the gene *TTC28* may be relevant for colon and rectal cancer. Although *TTC28* was briefly mentioned in the TCGA study [43] on colorectal cancer as being frequently hit by translocations, there was no previous report indicating biallelic inactivation of this gene by deletions and translocations. However, more evaluation is necessary to validate these hypotheses. Another recent study on colorectal cancer analyzed 92 samples using *whole genome sequencing* (WGS) and reported frequent L1 retrotranspositions originating from the first intron of *TTC28*[46]. These occur in the same narrow region as the translocations found in [43]. Thus the authors of [46] suggested that these genomic events reflect the same phenomenon and should be interpreted as L1 retrotranspositions, rather than translocations.

To communicate their findings, users can quickly export the images generated in *FISH Oracle 2* in different high quality formats. FISH Oracle is flexible regarding the underlying genome as long as genomic coordinates refer to the same genome assembly as the gene annotation.

We emphasize that *FISH Oracle 2* is tailored for visualizing *processed* genomic data sets that have been derived from raw data such as probe intensity values from microarray experiments or sets of NGS-reads that were already mapped to a reference genome. The focus on processed data leads to an important advantage: the processing step typically condenses several data points and abstracts from often irrelevant details of the data, so that different sets of processed data of the same kind become comparable. This fact is exploited by *FISH Oracle 2*, which provides a framework for simultaneous comparison of hundreds of samples. The approach of *FISH Oracle 2* to visualize processed data for many samples is complementing existing approaches to visualize raw data, as e.g. implemented in Trackster [10] or Savant2 [14]. However, visualization of raw data for *many* samples usually provides too many details cluttering the image, which in turn becomes too complex to be interpreted. As a consequence, raw data is usually only visualized for one or a few samples.

*FISH Oracle 2* has many features in common with generic genome browsers. Like these, *FISH Oracle 2* visualizes genome annotations and various kinds of genomic data on a linear scale defined by genomic coordinates. The main difference is that the generic genome browsers only display a fixed set of genomic events which have been retrieved from a database provided by the organization hosting the genome browser or stored in a file, uploaded by a user. The selection of specific subsets of the data has to be done separately, e.g. by tailored filter scripts. This can become cumbersome, given the many attributes according to which the data may be selected. In contrast, *FISH Oracle 2* provides a fully integrated search menu to specify the selection attributes interactively and to save them for later reuse.

Most of the widely used genome browsers like the UCSC and the Ensembl browser are provided on a web server hosted by publicly funded organizations. Uploading custom data to a server for visualization in the context of publicly available data may raise problems related to privacy concerns or server capacity, if the private data sets are very large. Of course, an alternative is to install the genome browser software on a private server. As there are often many software interdependencies and considerable hardware requirements for installing genome browsers, this option may not always be feasible for the average research group.

The described shortcoming of generic genome browsers and NGS browsers have been noticed previously and motivated the development of several special purpose software tools for visualizing processed genomics data. Of these software tools, *myKaryoView*[19] and the Gaggle genome browser [18] are most similar to *FISH Oracle 2*. All three software tools have several features in common, but each one has at least one unique feature (Table 1).

**Table 1:** Features of FISH Oracle 2, myKaryoView and the Gaggle genome browser

**Feature**	**Page number with explanation**	***FISH Oracle 2***	**myKaryo-View**	**Gaggle genome browser**
Unlimited number of tracks	6	*√*	*√*	*√*
Support of multiple genomes	5	*√*		*√*
Data filtering	4	**√**		
Saving of search configurations	7	**√**		
Relational database backend	3	**√**		**√**
High quality image export	6	**√**		
Data administration	7	**√**		
Tabbed browsing	6	**√**		
Grouping of segments	5	**√**		
Whole chromosome view	4	**√**	**√**	**√**
High resolution view	4	**√**	**√**	**√**
DAS support			**√**	
Links to external generic genome browser	6	**√**	**√**	**√**
Detailed feature information	5	**√**	**√**	
Bookmarks to genomic locations				**√**
Individual element coloring	6	**√**		**√**
Display of heatmaps				**√**
Integration of R				**√**

*FISH Oracle 2* and *myKaryoView* are web applications while the Gaggle genome browser is a desktop application. A bold checkmark is used for features unique among the three software tools. The second column shows the page number in which the corresponding *FISH Oracle 2* feature is explained.

*myKaryoView* focuses on the integration of personal genomics data from services like 23AndMe with publicly available reference data like COSMIC [47] or OMIM [48]. Retrieval of public and personal data can only be done via the DAS protocol [20]. This property is convenient in cases where the data is available on a DAS server. However, the user always has to set up a DAS server, even for their own personal data. Selection of data according to relevant attributes is not implemented in *myKaryoView*, so that one would have to implement filters applied to the DAS source to allow for selection of subsets of the data.

Compared to *FISH Oracle 2*, *myKaryoView* is less flexible with respect to the genome assembly the visualization refers to. If one wants to visualize data for a human genome assembly other than NCBI36 (which is the default and has been superseded by the latest assembly GrCh37 in mid 2009), the source code of *myKaryoView* needs to be changed. On the technical level, *myKaryoView* and *FISH Oracle 2* follow different approaches: *myKaryoView* transfers the data from the web server to the client and generates the visualizations locally in the browser. In contrast, *FISH Oracle 2* generates images on the server and transfers them to the client for display in the browser. Depending on the amount of data and the density of the images, one or the other approach may be advantageous. In practical scenarios, both tools quickly generate visualizations.

While *myKaryoView* integrates several publicly available data sources with one (private) high throughput genomic data set, *FISH Oracle 2* integrates different sets of (usually private) genomic data sets with a single publicly available data set (the genome annotation). The Gaggle genome browser (GGB, for short) follows a different data integration approach focusing on the integration of the *same* data at different stages of processing, from raw data to processed data derived from it. That is, for example, microarray probe intensity values can be visualized together with segment data derived from them. GGB is a desktop application which keeps all data locally in an SQLite database. A unique feature of GGB is the use of caching algorithms to minimize database access. This facilitates handling the large sets of raw data which are relevant for the visualization approach of GGB. On the other hand, a desktop application is less well suited for a distributed, collaborative research approach. Unfortunately, GGB only allows for interactive import of the data, sample by sample, via a graphical user interface, a process which quickly becomes cumbersome if many samples are to be imported. The number of tracks is generally unlimited. It is possible to load several samples into one track of the GGB visualization, but then the samples are not distinguishable any more. While strong copy number changes can easily be recognized, subtle changes often remain hidden, independently of the kinds of elements chosen for visualization (e.g. segments, lines, dots).

In contrast to *FISH Oracle 2*, the Gaggle genome browser can display the data in different modes, for example as data points, segments or heatmaps. Nevertheless, the presentations of several datasets containing hundreds of samples quickly becomes incomprehensible when displayed as a heatmap. A unique feature of GGB is the integration of data processing, data retrieval from sequence, interaction and pathway databases and data visualization. This becomes possible by connecting GGB to the Gaggle framework, see [18,22] for details.

Altogether *FISH Oracle 2* is designed to serve a specific purpose which is to compare different kinds of genomic data originating from large amounts of cancer tissue samples. Employing processed data which abstracts from many details of raw data allows to handle hundreds or even thousands of samples which would be impossible when using raw data. It is not intended that *FISH Oracle 2* competes with generic genome browsers. It rather provides capabilities to map own genomic data directly to genomic positions of genes, which for cancer research, are the most important genomic landmarks. Further kinds of annotations, e.g. regulatory features are intentionally not considered to avoid visualizations cluttered with too many details. If further annotation data needs to be shown, a single mouse click on the Ensembl or UCSC button in *FISH Oracle 2* redirects the user to the corresponding genome browser displaying the genomic region currently visualized in *FISH Oracle 2*.

At a technical level it would be desirable for *FISH Oracle 2* to provide more direct user interactions like drag and drop navigation or track reordering. This would however require advanced HTML5 web-technologies for the data visualization process. However, with the current state of technology, it is questionable, whether an implementation of a web browser visualization engine, based on such techniques, can efficiently generate displays of comparable quality and provide the full functionality of *FISH Oracle 2*. The challenges are manifold, requiring solutions to efficiently transfer the data to be visualized between server and client, and the choice of the appropriate HTML5 implementation providing the best compromise between user convenience and efficiency.

## Conclusions

We have developed a web server for the interactive visualization of downstream processed genomic data facilitating explorative data analysis. The interactive nature of *FISH Oracle 2* and the possibility to store, select and visualize large amounts of data supports life scientists in generating hypotheses from the visualization. The export of high quality images supports explanatory data visualization, simplifying the communication of new biological findings. Altogether, *FISH Oracle 2* complements existing software solutions by several novel features extending the current possibilities of genomic data visualization.

## Availability and requirements

To run *FISH Oracle 2* on the client side, a recent web browser with JavaScript support is required. For running the web server, a Linux system with at least one gigabyte of RAM is recommended. Hard disk requirements mainly depend on the size of the Ensembl database. A recent copy of the Ensembl *Homo sapiens* database requires about 13 gigabyte.

A *FISH Oracle 2* demo server, documentation and the software sources are available at http://www.zbh.uni-hamburg.de/fishoracle. The software is released under the ICS open source license. We have made some effort to keep the installation as easy as possible. We also provide a virtual machine image containing a ready to use instance of *FISH Oracle 2* and all necessary software dependencies. The demo server already contains a unique collection of data from several published prostate cancer studies as well as the data sets used for the two case studies.

## Competing interests

The authors declare that they have no competing interests.

## Authors’ contributions

RS and SK conceived the FISH Oracle project. MM and SK developed the software architecture. MM implemented the software and generated the results. All authors wrote and approved the final manuscript.

## Supplementary Material

Additional file 1 **Additional tables and figures.** The additional file 1 contains further tables and figures with visualizations of data from the ICGC and the TCGA project. Click here for file
