# Supplementary material for: FISH Oracle 2: a web server for integrative visualization of genomic data in cancer research
Source: J Clin Bioinforma. 2014 Mar 31;4:5. doi: 10.1186/2043-9113-4-5 (PMC4230720; doi:10.1186/2043-9113-4-5)
Supplement: Additional file 1 — Additional tables and figures. The additional file 1 contains further tables and figures with visualizations of data from the ICGC and the TCGA project. [file 2043-9113-4-5-S1.pdf]

FISH Oracle 2: A web server for integrative visualization of genomic data in cancer research

Additional file 1 — Additional tables and figures

Malte Mader, Ronald Simon and Stefan Kurtz

March 18, 2014

Table S1: Feature comparison between *FISH Oracle 1* and *FISH Oracle 2*.

Feature	*FISH Oracle 1*	*FISH Oracle 2*
display of Ensembl genes	$\surd$	$\surd$
selection of Ensembl database versions		$\surd$
application administration	$\surd$	$\surd$
image export	$\surd$	$\surd$
display of segments (CNV data)	$\surd$	$\surd$
assignment of status values to segment data		$\surd$
high quality image export	$\surd$	$\surd$
grouping of segment data		$\surd$
optional captions for segments		$\surd$
display of SNP data		$\surd$
display of translocation data		$\surd$
display of generic data		$\surd$
links to external sources		$\surd$
custom feature coloring		$\surd$
flexible track and filter system		$\surd$
storing of search configurations		$\surd$
comprehensive data administration		$\surd$
user profile view and administration		$\surd$
FISH Oracle importer		$\surd$

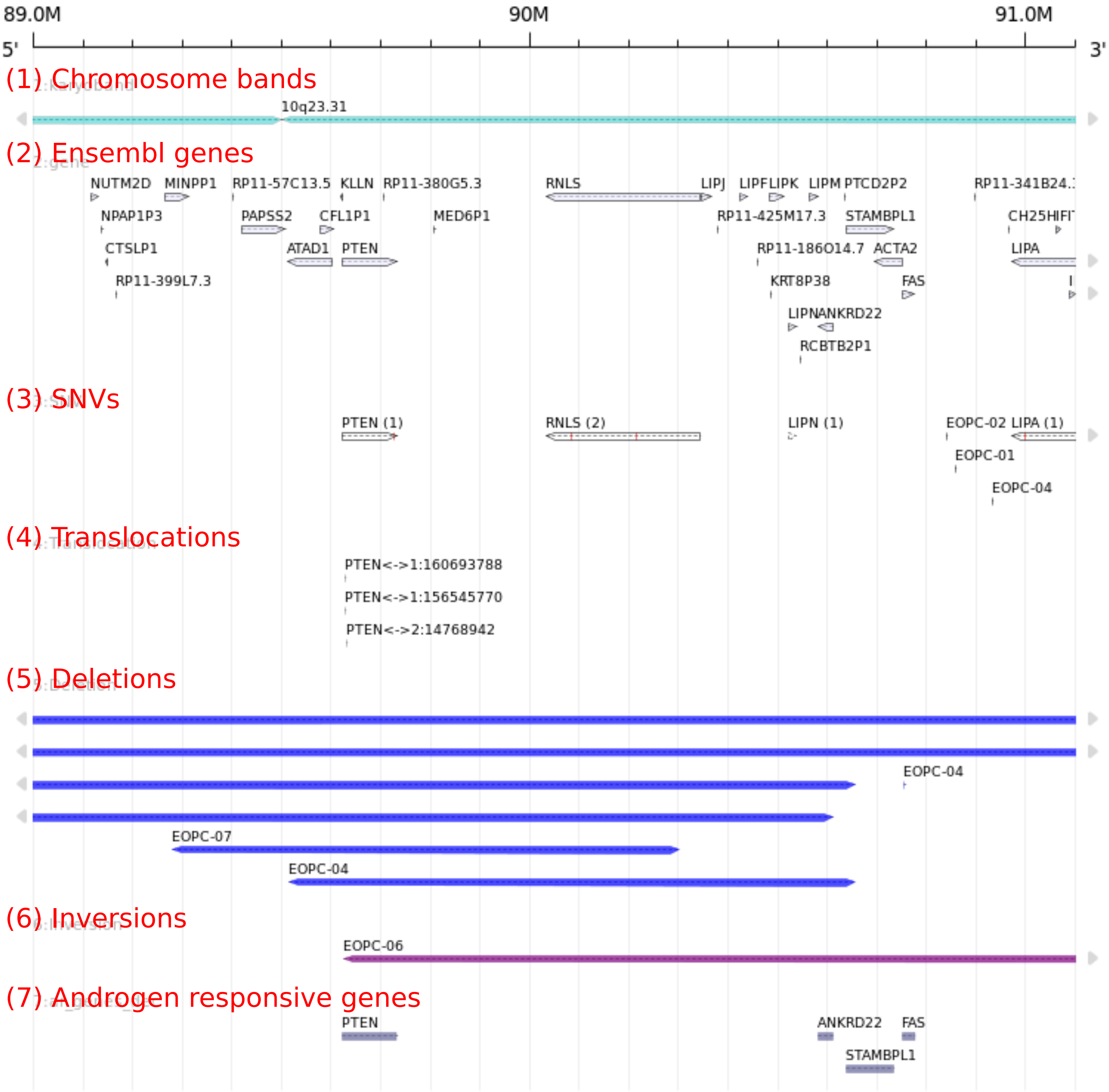

Figure S1: Genomic aberrations in EO-PCA at the 10q23 locus. This region focuses on the tumor suppressor gene *PTEN*. In addition to the chromosome band (1) and gene annotations (2), five data tracks (dataset "Weischenfeldt" in the demo application) are displayed. Track (3) shows all SNVs, one of which targets *PTEN*. Track (4) shows all translocations, three of which disrupt *PTEN*. Track (5) shows all deletions, illustrating the characteristic deletion pattern of *PTEN* in prostate cancer. Track (6) shows inversions. Track (7) shows a self compiled list of down regulated androgen responsive genes. These genes were experimentally defined by expression changes in the LNCAP cell line after treatment with androgen. Track 5 and 6 use generic elements.

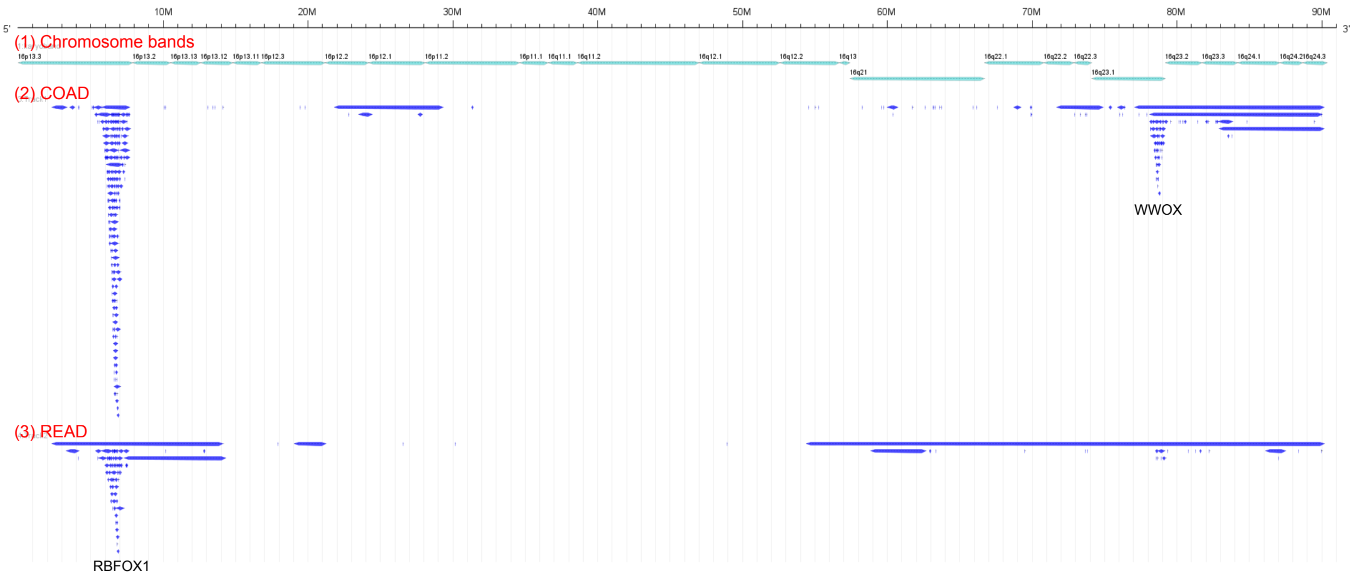

Figure S2: Comparison of colon (COAD) and rectal (READ) adenocarcinomas in an overview of the complete human chromosome 16. In addition to the human chromosome band in track (1), two data tracks of CNV intensity data at a threshold of -0.5 are shown. Track (2) displays data from colon samples and track (3) displays data from rectal samples. The visualization reveals deletions of the gene *RBFOX1* in both datasets and a deletion peak of the gene *WWOX* for colon data. The deletions of the rectal samples at the 16q23 locus are not as pronounced as the deletions of the colon samples.

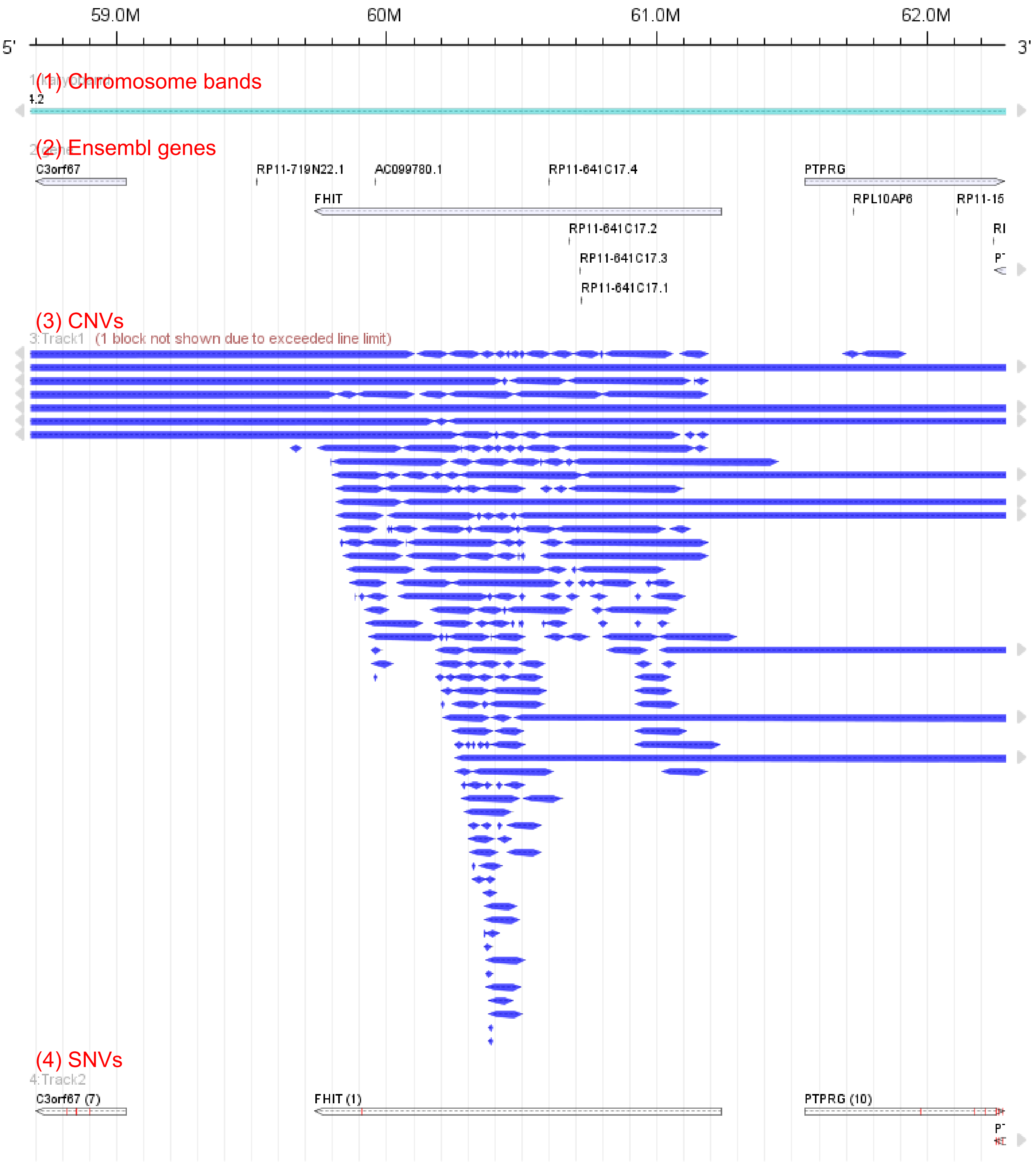

Figure S3: Genomic aberrations in colon and rectal tumors at the 3p14 locus. This region focuses on the tumor suppressor gene *FHIT*. In addition to the chromosome band (1) and gene annotations (2), two data tracks (datasets "TCGA_COAD" and "TCGA_READ" in the demo application) are shown. Track (3) shows CNV intensity data in form of deletions at a threshold of -0.5, most of which overlap with the gene *FHIT*. Track (4) shows one SNV affecting *FHIT*.

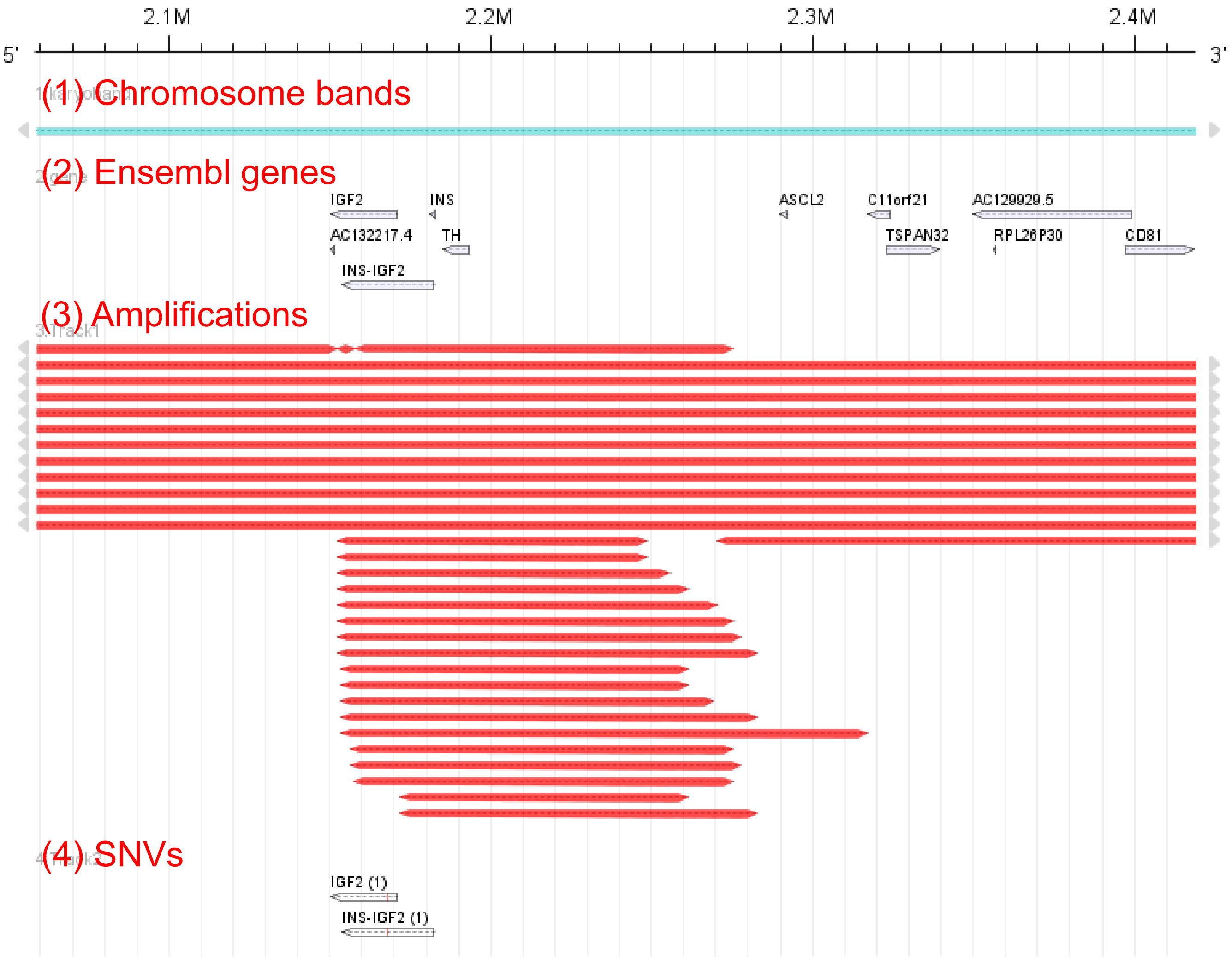

Figure S4: Amplifications in colon and rectal tumors at the 11p15 locus. This region focuses on the recently found amplification including the gene INS [1]. In addition to the chromosome band (1) and gene annotations (2), two data tracks (datasets "TCGA_COAD" and "TCGA_READ" in the demo application) are shown. Track (3) shows CNV intensity data in form of amplifications at a threshold of 0.25, most of which overlap with the gene *INS*. Track (4) shows two SNVs affecting *IGF2* and *INS-IGF2*, but not *INS*.

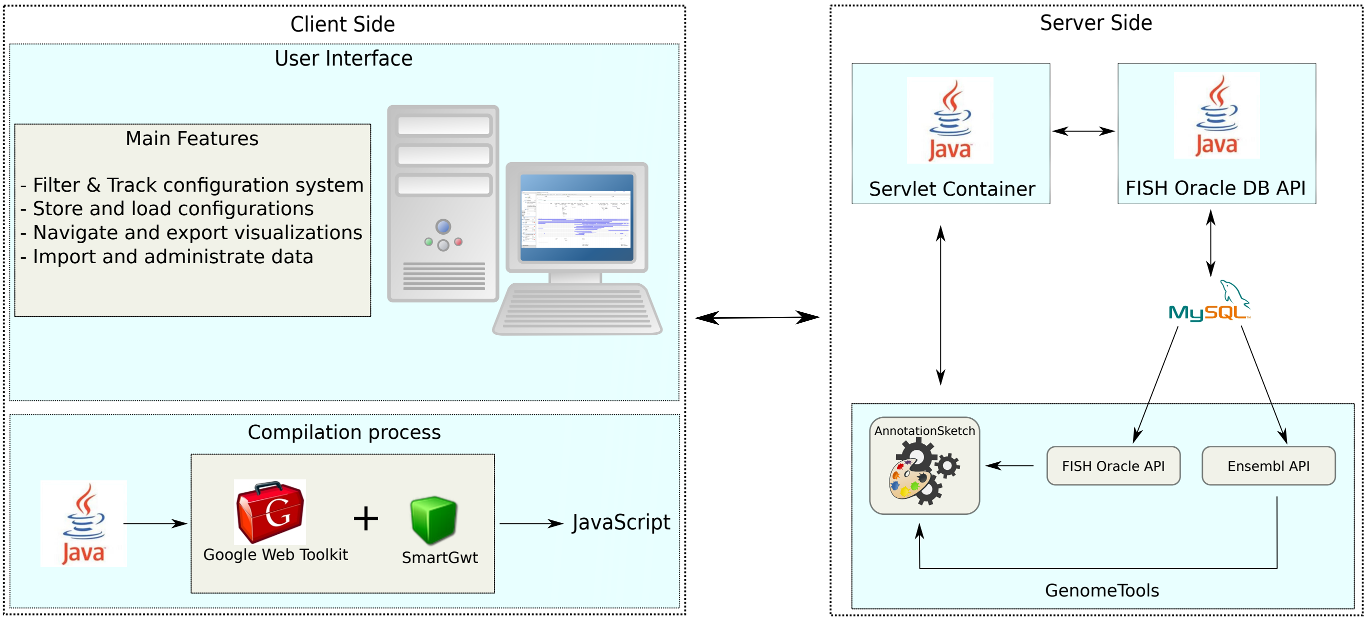

Figure S5: Diagram depicting the features and components of the client side and server side of *FISH Oracle 2*. The client side part shows the main features of *FISH Oracle 2* and the compilation process for the Java code. The program code on the client side is written in Java and then compiled to JavaScript by the Google Web Toolkit and SmartGWT. The JavaScript code then runs within the web browser providing the user interface with all necessary visualization and administration features. Data is transferred between the client and server side. At the server side Java servlet containers are responsible for data import, data extraction from and data modification within the FISH Oracle database. These processes are managed by the FISH Oracle database API. Data fetched by the FISH Oracle or Ensembl API is processed and visualized by AnnotationSketch. The resulting image containing the visualization and meta information including search configurations are passed back to the client side.

References

[1] Cancer Genome Atlas Network: **Comprehensive molecular characterization of human colon and rectal cancer.** *Nature* 2012, **487**(7407):330–337, [http://dx.doi.org/10.1038/nature11252].
